# Macrophage Exosomes Induce Placental Inflammatory Cytokines: A Novel Mode of Maternal–Placental Messaging

**DOI:** 10.1111/tra.12352

**Published:** 2016-01-26

**Authors:** Beth Holder, Tessa Jones, Vanessa Sancho Shimizu, Thomas F. Rice, Beverly Donaldson, Marielle Bouqueau, Karen Forbes, Beate Kampmann

**Affiliations:** ^1^ Section of Paediatrics, Division of Infectious DiseasesDepartment of Medicine, Imperial College LondonLondonUK; ^2^ Virology, Division of Infectious DiseasesDepartment of Medicine, Imperial College LondonLondonUK; ^3^ Division of Reproduction and Early DevelopmentLeeds Institute of Cardiovascular and Metabolic Medicine (LICAMM), The University of LeedsLeedsUK; ^4^ Maternal and Fetal Health Research Centre, Institute of Human DevelopmentThe University of ManchesterManchesterUK; ^5^ Vaccines & Immunity ThemeMRC UnitBanjulThe Gambia

**Keywords:** cytokines, endocytosis, exosomes, extracellular vesicles, immunology, macrophage, microvesicles, placenta, pregnancy, reproductive, trophoblast

## Abstract

Exosome trafficking from the placenta into the maternal circulation is well documented; the possibility that this trafficking is bi‐directional was unknown. We demonstrated clathrin‐mediated endocytosis of macrophage exosomes by the human placenta. We also demonstrated that macrophage exosomes induced placental production of cytokines interleukin (IL)‐6, IL‐8 and IL‐10. Exosomes therefore comprise an additional mechanism of immune cell signalling to the placenta, potentially facilitating protective responses to maternal inflammation and infection in pregnancy. This represents a novel mode of maternal–placental messaging.

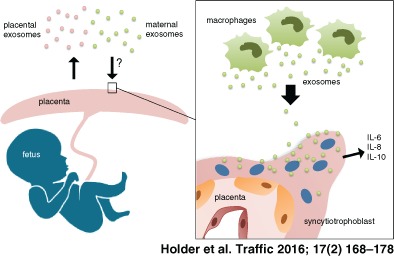

## Abstract (ab)

During pregnancy, the placenta forms the interface between mother and fetus. Highly controlled regulation of trans‐placental trafficking is therefore essential for the healthy development of the growing fetus. Extracellular vesicle‐mediated transfer of protein and nucleic acids from the human placenta into the maternal circulation is well documented; the possibility that this trafficking is bi‐directional has not yet been explored but could affect placental function and impact on the fetus. We hypothesized that the ability of the placenta to respond to maternal inflammatory signals is mediated by the interaction of maternal immune cell exosomes with placental trophoblast. Utilizing the BeWo cell line and whole placental explants, we demonstrated that the human placenta internalizes macrophage‐derived exosomes in a time‐ and dose‐dependent manner. This uptake was via clathrin‐dependent endocytosis. Furthermore, macrophage exosomes induced release of proinflammatory cytokines by the placenta. Taken together, our data demonstrates that exosomes are actively transported into the human placenta and that exosomes from activated immune cells modulate placental cytokine production. This represents a novel mechanism by which immune cells can signal to the placental unit, potentially facilitating responses to maternal inflammation and infection, and thereby preventing harm to the fetus.

Exosomes are small 30–150 nm vesicles formed within cells by inward budding of the limiting membrane of multivesicular bodies (MVBs) within the cytoplasm. Following fusion of MVBs with the plasma membrane of the cell, exosomes are released into the extracellular space. Originally described 30 years ago, exosomes have experienced a resurgence of interest in the wake of the studies implicating their role in multiple cell–cell signalling events, particularly in the immune system. Exosomes are released by many types of immune cells, including macrophages, dendritic cells and B cells 1, 2, 3 and play diverse roles, including mediating T cell activation 4, 5 and maturation of dendritic cells 6. Thus, exosomes are actively involved in immune cell communication.

The human placenta releases a large amount of sub‐cellular vesicles into the maternal circulation, including exosomes and the larger microvesicles. Placental microvesicles have often been demonstrated to be pro‐inflammatory 7, 8, 9, 10, whilst placental exosomes may be more important in inducing a tolerant immune response 11, 12, although this is not always the case. For example, both exosomes and microvesicles from placenta cause immunomodulation by altering toll‐like receptor (TLR)‐mediated responses of maternal immune cells 10, 13. Whilst the release of extracellular vesicles from the placenta is a normal process of pregnancy, elevated production of placental microvesicles and exosomes, and their heightened pro‐inflammatory effect on maternal immune cells are both implicated in pre‐eclampsia, a disorder of pregnancy characterized by systemic inflammation 7, 14.

There is growing appreciation of the importance of feto‐placental‐maternal crosstalk in establishing and maintaining healthy pregnancy. Whilst it is now widely accepted that placenta‐derived extracellular vesicles can influence the maternal immune response during pregnancy, bi‐directional trafficking between immune cells and the placenta has yet to be explored. This is particularly important because the placenta also mounts its own immunological responses to infection: TLRs, which recognize and respond to pathogen‐associated‐molecular‐patterns (PAMPs), are present in the human placenta throughout gestation 15, 16, and stimulation of these placental TLRs results in a robust cytokine response by trophoblast cells 11, 17. Virtually all cytokines have been detected in the human placenta at varying levels. In addition to placental cytokine responses driven by direct infection of the placenta 18, placental inflammatory responses can be observed in the absence of placental infection, although driven by signals from activated maternal cells 19, 20. Thus, the human placenta is an active immunological organ, which can respond both to infectious agents and to activation signals from the maternal immune system.

In light of evidence that various immune cells signal via exosomes, and that the placenta responds to maternal inflammatory signals, we hypothesise that the ability of the placenta to respond to inflammation can be mediated by uptake of immune cell exosomes. Here, we demonstrate that human trophoblast cells take up macrophage exosomes by an active endocytic mechanism and that this interaction impacts on placental pro‐inflammatory cytokine production. This reveals a novel mode of maternal to placental cellular messaging.

## Results and Discussion

### Isolation and characterization of THP‐1 macrophage exosomes

**Figure 1:**
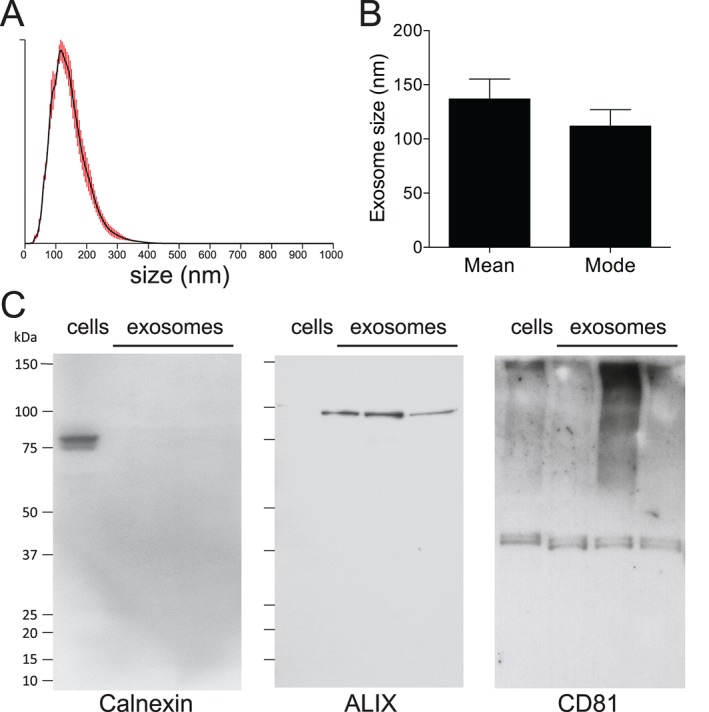
**Isolation of exosomes from PMA‐activated THP‐1 cells.** Exosomes were isolated by differential centrifugation from THP‐1 cells differentiated to macrophages by PMA treatment. Exosome phenotype was assessed by Nanosight Tracking Analysis (NTA) and western blotting for exosome markers. A) Representative NTA histogram demonstrating size distribution of exosome isolation from macrophages. Grey error bars indicate ±1 standard error of the mean. B) Mean and mode size of isolated macrophage exosomes, n = 8; C) Western blotting for negative exosome marker calnexin and positive exosome markers ALIX and CD81.

We utilized the THP‐1 monocyte‐derived macrophages as a model cell line for generation of immune cell exosomes as they can be readily differentiated into macrophages, and release high levels of exosomes. Analysis by Nanosight Tracking Analysis (NTA), an established model for sizing/counting extracellular vesicles 21, 22, demonstrated that vesicles isolated by a sequential centrifugation method had a mean size of 137.6 nM ± 18.50 and mode size of 111.9 ± 15.09 consistent with previously demonstrated size of exosomes (Figure 1A,B). Furthermore, isolated vesicles were positive for both the tetraspanin CD81, which is embedded in the exosome membrane and ALIX, localized in the lumen of exosomes, indicating the presence of intact exosomes (Figure 1C). Calnexin, a marker of endoplasmic reticulum, was only detectable in whole cell extracts, demonstrating that exosome isolates were not contaminated with cellular debris (Figure 1C). These macrophage‐derived exosomes were used in subsequent uptake experiments.

### Uptake of macrophage exosomes by human trophoblast cells

**Figure 2:**
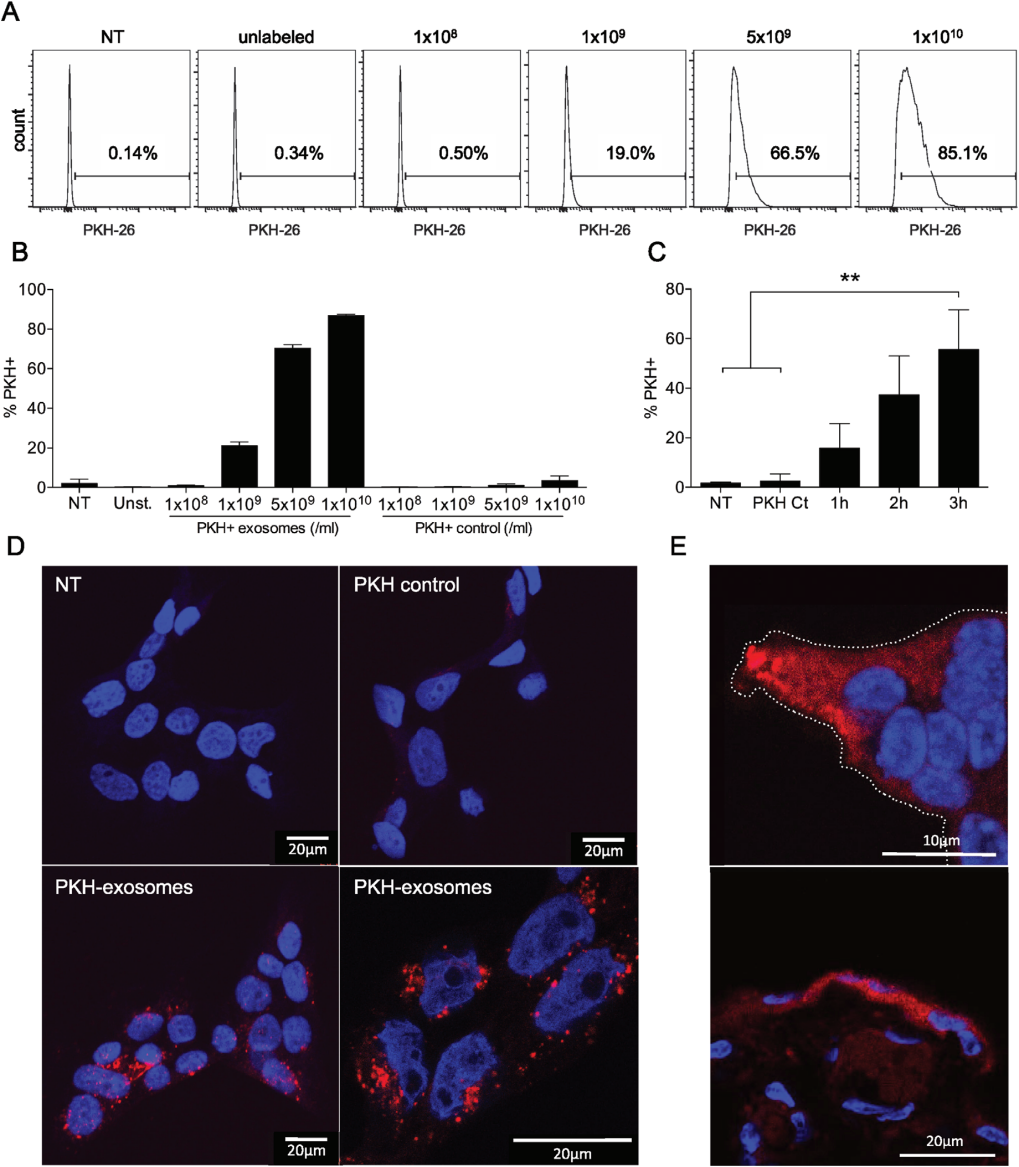
**Uptake of macrophage exosomes by trophoblast cells.** BeWos were incubated with PKH‐labelled exosomes, followed by trypsinization and flow cytometry. To control for possible flow‐through and passive uptake of dye, a control of PKH without exosomes was included, and added at the same volume as exosomes (PKH control/Ct). A) Representative histograms showing % PKH‐positive cells after 3 h incubation with increasing concentrations of PKH‐labelled exosomes, or the highest concentration of unlabelled exosomes (1x10^10^/ml; unlabelled). B) % PKH‐positive cells after 3 h incubation with increasing concentrations of exosomes, n = 4. C) % PKH‐positive cells after incubation with 5 × 10^9^ exosomes for 1, 2 or 3 h, n = 5. D) Confocal microscopy of BeWos following 3 h incubation with labelled exosomes (NT, non‐treated). E) Confocal microscopy term placental explants following 3 h incubation with labelled exosomes (villous tip indicated by dotted line).

To determine if placental trophoblast cells take up immune cell exosomes, we initially investigated macrophage exosome uptake kinetics in the BeWo cell line. BeWos are a choriocarcinoma cell line commonly used to investigate trophoblast biology. We demonstrated dose‐dependent, time‐dependent exosome uptake, with an increase in percentage of PKH‐positive BeWos by flow cytometry (Figure 2A–C). As BeWos were trypsinized and washed prior to flow cytometry, detectable fluorescence indicates the internalization of exosomes rather than just surface binding. Addition of unstained exosomes had no effect (Figure 2A). Confocal microscopy further demonstrated cellular association and uptake of PKH‐labelled macrophage exosomes into trophoblast cells (Figure 2D). We next moved to *ex vivo* culture of human placental explants, and demonstrated uptake of labelled exosomes into organized placental tissue, as visualized by confocal microscopy (Figure 2E, Movie S1, Supporting Information). Uptake was often concentrated in villous tips and was predominantly in the trophoblast cell layers. To rule out contamination with unbound PKH dye and non‐specific transfer to cells in these experiments, we utilized a PKH labeling control. This control was obtained during the PKH staining procedure by including a tube without exosomes to which PKH dye was added. Using all methods, no increased fluorescence was observed following incubation with the PKH labeling control, confirming specificity of detected exosome uptake. Vargas et al. 23 recently reported that BeWo‐derived exosomes can re‐enter BeWo cells. Our data comprise the first demonstration, to our knowledge, of the uptake of heterologous exosomes by the human placenta.

### Exosome uptake by human trophoblast cells is an active clathrin‐dependent endocytic process

**Figure 3:**
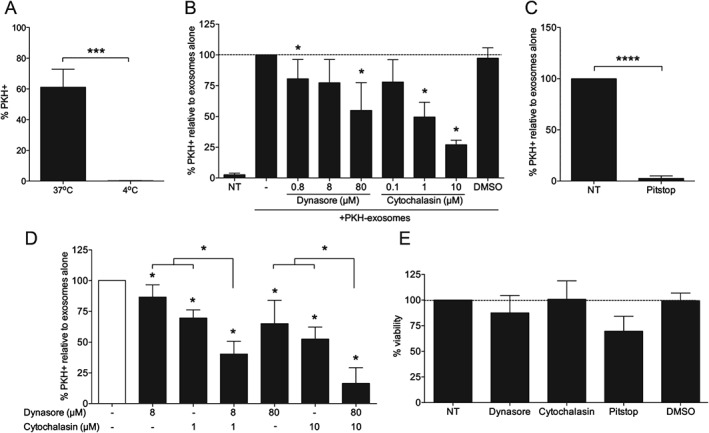
**Active placental uptake of macrophage exosomes by clathrin‐mediated endocytosis.** BeWos were incubated with 5 × 10^9^/mL PKH‐labelled exosomes for 3 h, followed by trypsinization and flow cytometry. A) % PKH+ BeWos following incubation with exosomes at 37 °C or 4 °C, n = 7. B) Exosome uptake following 30 min pre‐treatment with endocytosis inhibitors dynasore or cytochalasin, reported as % of exosome treatment alone (−), n = 6, B) Exosome uptake following 30 min pre‐treatment with endocytosis inhibitors dynasore or cytochalasin D, reported as % of uptake in the absence of treatment, n = 6. C) Exosome uptake following 15 min pre‐treatment with 25 μM Pitstop 2 before addition of exosomes for 30 min, reported as % of uptake in the absence of treatment, n = 5. D) Exosome uptake following 30 min pre‐treatment with endocytosis inhibitors dynasore or cytochalasin D alone and in combination, reported as % of uptake in the absence of treatment, n = 6. B,C) Percent cell viability following treatment with endocytosis inhibitors, relative to no treatment, n = 3.

At 4 °C, there was complete abolition of exosome uptake in BeWo cells (Figure 3A), demonstrating that uptake was an active process. To determine if endocytosis was the route of exosome entry, as previously shown in other cell types 24, 25, cells were pre‐incubated with endocytosis inhibitors cytochalasin D or dynasore prior to addition of labelled exosomes. Cytochalasin D causes de‐polymerization of the actin cytoskeleton, thereby inhibiting multiple endocytic pathways. Dynasore is a specific small molecule inhibitor of dynamin activity 26. Dynamin is considered a master regulator of endocytosis, being required for all but some clathrin‐ and caveolae‐independent endocytoic uptake and has recently shown to inhibit endocytic uptake of exosomes into epithelial cells 27. The highest concentration of dynasore reduced exosome uptake by 45% (p < 0·05, Figure 3B). The highest concentration of cytochalasin D reduced uptake by 72% (p < 0·05). This demonstration of the role of endocytosis in exosome uptake by the placenta is similar to studies investigating uptake of Epstein–Barr virus (EBV)‐infected B cell exosomes by epithelial cells 27, oligodendrocyte exosomes by microglia 28 and reticulocyte exosomes by macrophages 29. Combination treatment with dynasore and cytochalasin D resulted in synergistic reduction of exosome uptake, with 84% inhibition at the highest combined concentrations (Figure 3D). No effect was observed with the carrier control, dimethyl sulfoxide (DMSO), and no significant change in cell viability was observed following treatment with dynasore nor cychalasin D (Figure 3E). This data suggests there was both dynamin‐dependent and ‐independent exosome uptake by placental trophoblast.

This endocytosis could be either clathrin‐ or calveolin‐mediated. As human syncytiotrophoblast, which covers the maternal side of the placenta, expresses clathrin but not caveolae 30, 31, it was most likely that clathrin‐mediated rather than calveolin‐mediated endocytosis was the pathway for exosome uptake. Clathrin‐mediated endocytosis is involved in uptake of other molecules into the placenta such as albumin and cholesterol 31, 32. Using Pitstop 2, a cell‐permeable clathrin inhibitor, flow cytometric analysis showed complete abolition of exosome uptake, demonstrating that it was indeed clathrin‐dependent (Figure 3C). Pitstop treatment resulted in a small decrease in cell viability (Figure 3E), so for flow cytometric analysis of exosome uptake, it was ensured that only live cells, excluding DAPI, were gated. Therefore, uptake of exosomes by the placenta occurs via clathrin‐mediated endocytosis; the receptor for this uptake is under investigation.

### Macrophage exosomes induce pro‐inflammatory cytokine production by the placenta

Our data thus far indicates that macrophage exosomes are actively endocytosed into placental tissue. We sought to explore whether this interaction had any impact on placental function. As we utilized macrophages exosomes, this current study has particular implications for conveyance of signals from maternal immune cells to the placenta. Given the macrophage origin of our exosomes, and the ability of trophoblast to respond to inflammatory milieu, we examined the production of cytokines by the placenta. Initially we utilised BeWo cells, and found a significant induction of IL‐6 by macrophage exosomes (data not shown). However, we also found that our BeWo cells did not respond well to our positive control (1–100 ng/ml LPS), similar to previous studies which show that, despite expressing TLR‐4, BeWos do not respond as expected to LPS 33. Therefore, all subsequent functional experiments were performed only in the more relevant human placental explant model.

**Figure 4:**
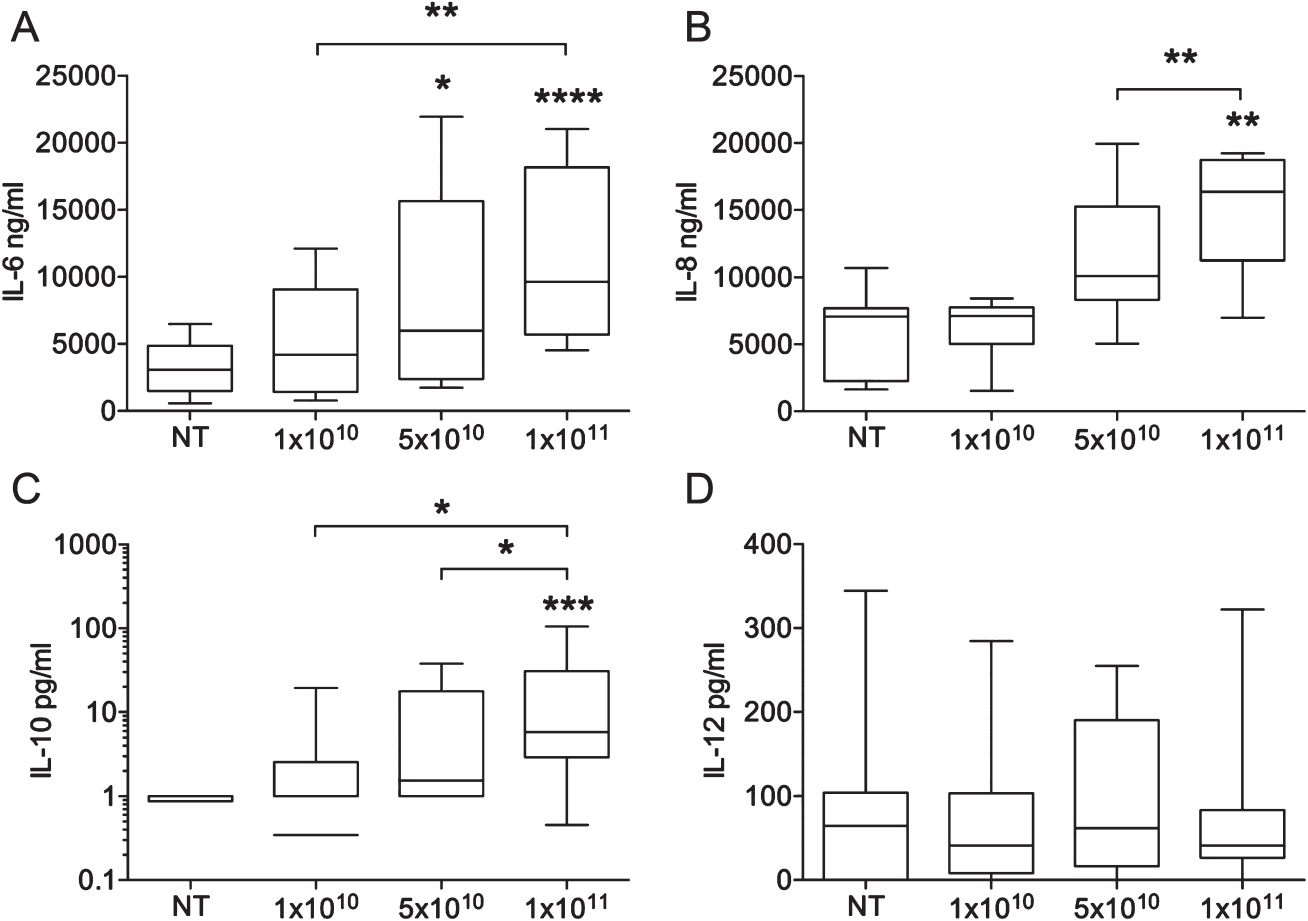
**Placental production of IL‐6**, **IL‐8, IL‐10 and IL‐12 in response to macrophage exosomes.** Placental explants were incubated with 1 × 10^10^–1 × 10^11^/mL exosomes for 24 h. Supernatants were collected and cytokines assayed by ELISA. Exosomes induced significant increases in the production of A) IL‐6 (n = 10), B) IL‐8 (n = 7), C) IL‐10 (n = 9) (all p < 0.0005, Friedman anova), but no increase in D) IL‐12 (n = 11, p = 0.95). Asterisks above individual columns indicates a significant difference from the non‐treated (NT), and differences between other columns are indicated by brackets (Dunn's multiple comparisons posthoc test).

Following 24 h incubation with macrophage exosomes, placental release of IL‐6 was significantly increased, with a 3·5‐fold induction at the highest concentration of 1 × 10^11^/mL exosomes (Figure 4A). Placental release of IL‐8 was also increased 2·4‐fold at the highest concentration of exosomes (Figure 4B). IL‐10 release was very low, with levels often undetectable in non‐treated wells; however, it followed a pattern similar to IL‐6/8, with significant increased release following incubation with macrophage exosomes (Figure 4C). IL‐12 was readily detectable in explant supernatants, but levels were more heterogenous than IL‐6/8 and there was no change following incubation with macrophage exosomes (Figure 4D). IFN‐γ, TNF‐α and IL‐17A were all undetectable in the explant supernatants (<40 pg/mL IFN‐γ, *n* = 5; <40 pg/mL TNF‐α, *n* = 12; <4 pg/mL IL‐17A, *n* = 2). Therefore, in summary, macrophage exosome induce placental release of IL‐6, IL‐8 and IL‐10.

Many studies have studied placental cytokines *in situ* at the mRNA and protein level, where a complete range of cytokines are expressed 34, but less have explored the profiles of cytokines released from explants *ex vivo*. Although the placenta contains some cytokine‐producing immune cells, the majority of cytokines released into the maternal circulation come from the trophoblast layers. These specialized epithelial cells have been reported to release a cytokine repertoire similar to other epithelial cell types, including high IL‐6/IL‐8 production, which increases under inflammatory stimuli 35, 36, 37, 38. The increased release of IL‐8 in response to macrophage exosomes could be important in recruiting maternal immune cells to the maternal–foetal interface. Our results do differ from other studies that report resting TNF‐α release from placental explants 35, which we did not observe. The reason for this is unclear, but one factor that impacts on placental cytokines is the mode of delivery, and whilst our study utilized placentas from vaginal delivery, in that study, all placentas were from caesarean sections. Immunohistochemical measurement of *in situ* placental cytokine expression, in response to immune cell exosomes could provide more detailed information on the immunomodulatory effect of macrophage exosomes.

As macrophage exosomes can contain endogenous cytokines 39, we measured the IL‐6 and IL‐8 levels in our exosomes to confirm that the levels we were measuring in supernatants were due to release from the placenta. As seen in Figure S1, at the highest concentration of 1 × 10^11^/mL, the exosomes contained both IL‐6 (0·36 µg/mL ± 0·24), and IL‐8 (14·8 µg/mL ± 13·3). IL‐8 was surprisingly high, and could play a role in mediating placental responses to macrophage exosomes. However, for both cytokines, endogenous exosome levels could not account for the levels we measured in explant‐containing wells (Figure S1). Therefore the greatly increased levels we saw from explants following treatment with exosomes was due to *de novo* cytokine release from the placenta.

Our demonstration that macrophage exosomes stimulate placental cytokine release is supported by previous studies showing that exosomes derived from activated macrophages are taken up and cause activation of naïve recipient immune cells 3 and exosomes isolated from body fluids activate monocytes through TLRs 40. Furthermore, a series of papers from Schorey et al. have demonstrated that macrophages infected with intracellular bacteria, release exosomes containing PAMPs, which can activate naïve macrophages and CD4/CD8 T cells *in vitro* and *in vivo* 3, 41, 42. Conversely, this group found that exosomes released by cells infected with *Mycobacterium tuberculosis* suppress IFN‐γ‐mediated activation of naïve macrophages, helping the persistence of infection 43. Therefore, the exosome pathway represents an additional non‐cell‐associated mechanism of antigen transfer between immune cells, which can exert varying effects on naïve cells. We therefore hypothesise that macrophage‐derived exosome entry into the placenta could result in immunomodulation and alterations in the ability of the placenta to respond to subsequent inflammatory stimuli.

## Concluding remarks

Several studies have investigated the role of placental microvesicles and exosomes in modulation of maternal immune cells 7, 9, 10, 13. The converse interaction from mother to placenta has not been explored previously, but our data shows there is active endocytosis of heterologous exosomes by the human placenta. Moreover, we demonstrate here that the placenta can respond to these non‐contact‐dependent messages from activated macrophages, via exosomes, and therefore the exosome pathway may mediate protective placental immune responses during pregnancy. This study thereby reveals the proof of concept for a novel route of communication between the mother and placenta, via circulating exosomes.

## Materials and Methods

### Cell culture

BeWo cells were maintained in DMEM:F12‐Glutamax supplemented with 10% foetal calf serum (FCS) and antibiotics. THP‐1 cells were maintained in RPMI‐Glutamax supplemented with 10% FCS and antibiotics. For differentiation into macrophage cells, THP‐1 cells were seeded at 0**·**5–1 × 10^6^/mL with 50 ng/mL Phorbol 12‐myristate 13‐acetate (PMA) for 24 h, washed and changed to new media to rest for 48 h before starting exosome collection. For the exosome collection period, cells were cultured in media supplemented with 10% exosome‐depleted FBS (Exo‐FBS; System Biosciences) for 48–72 h.

### Placenta samples

Placentas were collected from healthy term pregnancies following vaginal delivery or caesarean section (37–42 week). NRES ethical approval (13/LO/1712) and written informed consent were obtained. Villous tissue was excised from three randomly selected areas of the placenta, washed in sterile PBS, and dissected in DMEM/F12 (Gibco) containing 2 mM l‐glutamine, 100 U/mL streptomycin and 100 U/mL penicillin (GSP) (Gibco).

### Exosome preparation and quantification

Exosomes were purified from cell culture supernatants by sequential centrifugation; 10 min at 1000 g to remove cells, 10 000 × ***g*** for 10 min to remove cell debris and then exosomes were pelleted at 100 000 × ***g*** for 90 min. The final pellet was resuspended in PBS or diluent C (for PKH‐staining, Sigma) and passed through a 0·2 μM filter. Exosomes were quantified and sized using Nanoparticle Tracking Analysis (NTA; LM10; Nanosight) following the manufacturer's instructions. Samples were loaded using a syringe pump to improve accuracy. Three videos of 90 s each were collected and batch analysed using the instrument software (NTA 2·3).

### Fluorescence labeling of exosomes

For labeling experiments, exosome pellets were resuspended directly in diluent C from the lipophilic PKH‐26 labeling kit (Sigma). Labelling was performed following the manufacturer's instructions, with the following modifications for exosome labelling. To stop the reaction, 2% BSA was used, as FBS contains endogenous exosomes. To remove unbound dye, preparations were loaded on to a 7 kDa Zeba spin column (Pierce). A PKH labelling control was obtained by putting a tube with diluent alone through the PKH staining procedure. This was used in subsequent experiments by adding the same volume as required for the concentration of the corresponding exosome sample (designated PKH control).

### Western blotting

Total protein extracts were prepared using RIPA buffer containing protease inhibitors and quantified by BCA assay (Pierce). Protein was prepared in NuPage LDS buffer plus β‐mercaptoethanol and heated at 95°C for 5 min. For CD81, protein was prepared in LDS buffer without β‐mercaptoethanol. Equal amounts of protein (20 µg) were separated on Bis‐Tris gels and transferred onto PVDF membrane (iBLOT gel transfer stacks; Invitrogen). Following protein block (1% BSA/5% skimmed milk), membranes were probed with primary antibody followed by incubation with HRP‐conjugated secondary antibodies. Antibodies against the following antigens were used: CD81 (RabMab, Abcam), ALIX (RabMab, Abcam), calnexin (Cell Signalling Technology). Secondary antibody was from Dako (Goat anti‐rabbit‐HRP). Finally, membranes were developed using ECL Prime Western blotting detection reagent (Amersham) and Hyperfilm (Amersham).

### BeWo exosome uptake assays

BeWo cells were plated on glass coverslips (for microscopy) or 12‐well cell culture plates (for flow cytometry) at 0·1 × 10^6^cells/mL and left to adhere overnight. Cells were washed and isolated THP‐1‐derived exosomes were added to cells at 10^9^–10^11^/mL (concentration determined by NTA) and incubated at 37°C. To determine if the uptake of exosomes was via an active process, cells were alternatively incubated at 4°C. For endocytosis inhibition, cytochalasin D (0·8–80 μM; C2618; Sigma) or dynasore (0·1–10 μM; D7693; Sigma) were added to cells 30 min before addition of exosomes. Following the results of these experiments, combination treatment with both inhibitors was also tested. The two higher doses of both dynasore (1 μM and 10 μM) and cytochalasin D (8 μM and 80 μM) were added to cells alone and in combination, 30 min before addition of exosomes. For clathrin‐inhibition, cells were treated with Pitstop 2 (25 μM; Abcam) under serum‐free conditions for 15 min prior to exosome addition for a further 30 min. Carrier controls were included for all experiments.

### Explant culture

Small placental villous explants (2–3 mm) were dissected in serum‐free DMEM/F12 containing 2 mM l‐glutamine, 100 U/mL streptomycin, and 100 U/mL penicillin (GSP) (Gibco). Explants were placed one per well, in a 96 well plate. To account for variation in explant size, treatments were performed in triplicate and randomized across the plate. Isolated exosomes were added to explants at 10^10^–10^11^/mL. To check for endogenous exosomal cytokines, exosome‐only control wells were incubated at the highest concentration alongside placental explants and included in subsequent ELISAs. Supernatants were collected after 24 h for cytokine analysis and explants were washed and fixed with 4% paraformaldehyde for microscopy.

### Flow cytometry

Following incubation with labelled exosomes, BeWo cells were washed, trypsinized, washed in media containing 5 mM EDTA and finally resuspended in FACS buffer (1% FCS, 5 mM EDTA in PBS). DAPI was added prior to acquisition for gating of viable cells. Samples were measured on either: a Becton Dickinson FortessaLSR equipped with 20 mW 355 nm, 50 mW 405 nm, 50 mW 488 nm, 50 mW 561 nm, 20 mW 633 nm lasers and a ND1·0 filter in front of the FSC photodiode or a Becton Dickinson FortessaLSR equipped with 50 mW 405 nm, 50 mW 488 nm, 50 mW 561 nm, 20 mW 633 nm lasers and a ND1·0 filter in front of the FSC photodiode. Acquisition was set to record 10 000 single cells, following FSC‐A/W, SSC‐A/W gating to exclude doublets and DAPI‐negative gating to exclude dead cells. PMT voltages were adjusted after standardized CST checks minimizing the spectral overlap to increase data precision. To detect PKH‐26 fluorescence, a 561 nM laser with 582/15 nM band pass filter was used. Analysis was performed using FlowJo.

### Confocal fluorescence microscopy

Following incubation with labelled exosomes, BeWo cells plated on glass coverslips were washed 3 times with PBS, fixed in 4% paraformaldehyde and mounted with Prolong Gold. Placental explants were washed and fixed before embedding in OCT, cryosectioning and mounting with Prolong Gold. Confocal images of BeWos were acquired on a Zeiss Pascal system. Confocal images of placental explants were acquired on a Leiss LSM 510 system. Image processing was performed using the Zeiss LSM image browser software and NIH ImageJ (http://rsbweb.nih.gov/ij; Open Source).

### Viability assays following endocytosis inhibition

Cytotoxicity of endocytosis inhibitors was tested using the CellTiter Aqueous One assay according to the manufacturers instructions (Promega). Cells were plated in duplicate in a 96 well plate at 0·1 × 10^6^/mL, 100 μL per well and incubated overnight. Following treatment with dynasore or cytochalasin D, 20 μL Cell Titer reagent was added to each well and the plate incubated at 37°C for 2 h followed by optical density measurement at 490 nm. ODs of treated cells were compared with the non‐treated cells, expressed as percentage viability.

### ELISAs

Culture supernatants were assayed for the following cytokines; IL‐6, IL‐8, IL‐10 and IL‐17A using commercial ELISA kits (IL‐6/IL‐8/IL‐17 Ready‐SET‐Go!; eBioscience, IL‐10 Diaclone) and IL‐12, IFN‐γ and TNF‐α using in‐house ELISAs (capture and biotinylated detection antibodies from BD, development with OPD). Sensitivities were as follows: 2 pg/mL for IL‐6, IL‐8, IL‐17A, 4·9 pg/mL for IL‐10, and 20 pg/mL IL‐12, IFN‐γ and TNF‐α. For IL‐10, IL‐17A, IFN‐γ and TNF‐α, for which levels were very low, the lowest dilutions performed were 1:2, meaning that levels reported as ‘undetectable’ were those below 4 pg/mL for IL‐17A and 40 pg/mL for IFN‐γ and TNF‐α.

### Statistical analysis

Following testing for Gaussian distribution using D'Agostino–Pearson omnibus normality test, appropriate parametric/non‐parametric tests were selected. For comparison of multiple groups, the Friedman test was performed, with Dunn's *post hoc* comparing all groups to the non‐treated. For the comparison of two groups, the Mann–Whitney *U*‐test was used. For inhibition experiments, Wilcoxon signed‐rank test was performed, comparing exosome uptake following treatment with inhibitors to uptake of exosomes without drug treatment (designated as 100%). All analysis was performed using graph pad prism 6.

## Supporting information


**Movie S1**: **Z stack of villous tip of placental explant showing uptake of labelled (red) exosomes into trophoblast cells (nuclei in blue)**.

**Figure S1**: **Endogenous exosomal IL‐6 and IL‐8**. Control wells of exosomes alone were incubated alongside placental explants at the highest concentration, and assayed for (A) IL‐6 (n = 4) and (B) IL‐8 (n = 3) by ELISA.
