# Supplementary material for: Macrophage Exosomes Induce Placental Inflammatory Cytokines: A Novel Mode of Maternal–Placental Messaging
Source: Traffic. 2016 Jan 26;17(2):168–78. doi: 10.1111/tra.12352 (PMC4738478; doi:10.1111/tra.12352)
Supplement: Supplementary file 2 — Figure S1: Endogenous exosomal IL‐6 and IL‐8. Control wells of exosomes alone were incubated alongside placental explants at the highest concentration, and assayed for (A) IL‐6 (n = 4) and (B) IL‐8 (n = 3) by ELISA. [file TRA-17-168-s002.docx]

**Macrophage exosomes induce placental inflammatory cytokines: a novel mode of maternal-placental messaging**

Running title- Exosome trafficking from the macrophage to the placenta

**Authors:**

Beth S. Holder^1*^, Tessa Jones^1^, Vanessa Sancho Shimizu^1,2^, Thomas F Rice^1^, Beverly Donaldson^1^, Marielle Bouqueau^1^, Karen Forbes^3,4^, Beate Kampmann^1,5^

**Affiliations:**

*1 Section of Paediatrics, Division of Infectious Diseases, Department of Medicine, Imperial College London*

*2 Virology, Division of Infectious Diseases, Department of Medicine, Imperial College London*

*3 Division of Reproduction and Early Development, Leeds Institute of Cardiovascular and Metabolic Medicine (LICAMM), The University of Leeds*

*4 Maternal and Fetal Health Research Centre, Institute of Human Development, The University of Manchester*

*5 Vaccines & Immunity Theme, MRC Unit, The Gambia*

*Correspondence to: Dr .Beth Holder, [b.holder@imperial.ac.uk](mailto:b.holder@imperial.ac.uk)

**Corresponding author contact details:**

Dr .Beth Holder

Paediatrics, 2^nd^ Floor, Wright-Fleming Building, St. Mary’s Campus, W2 1PG

0207 594 2063

[b.holder@imperial.ac.uk](mailto:b.holder@imperial.ac.uk)

Supplementary Materials:

**Movie S1** Z stack of villous tip of placental explant showing uptake of labeled (red) exosomes into trophoblast cells (nuclei in blue).

**Figure S1 Endogenous exosomal IL-6 and IL-8.** Control wells of exosomes alone were incubated alongside placental explants at the highest concentration, and assayed for A) IL-6 (n=4) and B) IL-8 (n=3) by ELISA.
